# JNK/p66Shc/ITCH Signaling Pathway Mediates Angiotensin II-induced Ferritin Degradation and Labile Iron Pool Increase

**DOI:** 10.3390/nu12030668

**Published:** 2020-02-29

**Authors:** Andżelika Borkowska, Urszula Popowska, Jan Spodnik, Anna Herman-Antosiewicz, Michał Woźniak, Jędrzej Antosiewicz

**Affiliations:** 1 Department of Bioenergetics and Physiology of Exercise, Medical University of Gdansk, 80-211 Gdansk, Poland; 2 Department of Medical Chemistry, Medical University of Gdansk, 80-211 Gdansk, Poland; urszula.popowska@gumed.edu.pl (U.P.); michal.wozniak@gumed.edu.pl (M.W.); 3 Department of Anatomy and Neurobiology, Medical University of Gdansk, 80-211 Gdansk, Poland; jan.spodnik@gumed.edu.pl; 4 Department of Medical Biology and Genetics, University of Gdansk, 80-308 Gdansk, Poland; anna.herman-antosiewicz@biol.ug.edu.pl

**Keywords:** free radical, iron, angiotensin II

## Abstract

Angiotensin II (Ang II) induces deleterious changes in cellular iron metabolism and increases the generation of reactive oxygen species. This leads to an impairment of neuronal and vascular function. However, the mechanism underpinning Ang II-induced changes in iron metabolism is not known. We hypothesized that Ang II-induced ferritin degradation and an increase in the labile iron pool are mediated by the c-Jun N-terminal kinase (JNK)/p66Shc/ITCH signaling pathway. We show that Ang II treatment induced ferritin degradation in an endothelial cell lines derived from the bovine stem pulmonary artery (CPAE), human umbilical vein endothelial cells (HUVEC), and HT22 neuronal cells. Ferritin degradation was accompanied by an increase in the labile iron pool, as determined by changes in calcein fluorescence. The JNK inhibitor SP600125 abolished Ang II-induced ferritin degradation. Furthermore, the effect of Ang II on ferritin levels was completely abolished in cells transfected with vectors encoding catalytically inactive variants of JNK1 or JNK2. CPAE cells expressing inactive ITCHor p66Shc (substrates of JNK kinases) were completely resistant to Ang II-induced ferritin degradation. These observations suggest that Ang II-induced ferritin degradation and, hence, elevation of the levels of highly reactive iron, are mediated by the JNK/p66Shc/ITCH signaling pathway.

## 1. Introduction

Angiotensin II (Ang II) regulates blood pressure and fluid balance by coordinating the activity of the renal, cardiovascular, and central nervous systems [1]. Ang II is synthetized in many different tissues, including the brain, kidney, heart, and vessels [2]. In addition, Ang II is involved in many brain activities, including memory acquisition and consolidation [3]. Disturbance of the renin-Ang II system in the brain is associated with anxiety, depression, and cognitive and emotional stress [4]. The molecular mechanisms of Ang II toxicity include an increase in amyloid plaque deposition and induction of oxidative stress, inflammation, and vascular damage [5,6]. 

Vascular diseases are associated with an increase in the production of reactive oxygen species (ROS) in the vessel wall and with endothelial dysfunction. It is generally assumed that increased ROS levels, particularly superoxide (O_2_^.–^) levels, on the one hand reduce nitric oxide (NO) bioavailability and on the other give rise to deleterious peroxynitrite. Ang II is a well-known inducer of endothelial cell activation and smooth muscle cell proliferation resulting from ROS-induced signal transduction [7,8]. ROS production by nicotinamide adenine dinucleotide phosphate oxidase (NADPH oxidase) and the activation of redox-dependent signaling cascades by Ang II are considered critical processes underlying neuronal-vascular injury and inflammation [9,10]. Further, an increasing number of reports demonstrate that Ang II induces iron accumulation in several tissues, including the aorta and neuronal tissue [11]. This might play a role in the impairment of vascular function and arterial remodeling induced by Ang II, possibly by enhancing iron-dependent oxidative stress [12]. In addition, iron chelation suppresses Ang II-induced upregulation of TGF-β1 in the heart and attenuates vascular dysfunction in the aorta [13,14].

The majority of intracellular iron is stored by ferritin, a protein composed of L and H subunits. Ferritin-stored iron is considered to be safe (inert), as it does not participate in free radical-generating reactions, e.g., the Fenton reaction and others. Hence, the iron-dependent formation of ROS is associated with the levels of free iron bound to low-molecular weight compounds, such as nucleotides and amino acids, etc. This iron constitutes the labile iron pool (LIP). LIP increase not only influences ROS formation but also induces changes in gene expression, also that of ferritin L and H genes and transferrin receptor and ferroportin genes. Therefore, LIP increase induces several adaptive changes to decrease the free iron levels and iron-dependent ROS formation. Ang II induces several changes in gene expression that can promote cell death or increase cell proliferation, and ROS mediates most of these changes. As mentioned above, the role of iron in Ang II-induced toxicity has been recognized; however, the molecular mechanism involved in this process is not known [15].

Ang II also activates stress-activated protein kinases (SAPK) including c-Jun N-terminal kinase (JNK) and the adaptor protein p66Shc [16,17]. Both SAPK and p66Shc are involved in ferritin degradation and LIP increase [18,19,20]. Furthermore, lysosomal and proteasomal ferritin degradation is observed in cells under stress conditions. The effect of Ang II on ferritin stability in the cell has not been determined to date. Consequently, the current study was undertaken to test the hypothesis that ferritin-bound iron is not inert in neuronal and endothelial cells treated with Ang II. We also aimed to re-examine the role of SAPK, ubiquitin ligase ITCH, and the adaptor protein p66Shc in Ang II-induced ferritin degradation.

## 2. Experimental Section

### 2.1. Cell Culture

Human umbilical vein endothelial cells (HUVEC, 200-05N) were purchased from the European Collection of Authenticated Cell Cultures (Porton Down, UK) and cultured in endothelial cell growth medium (211–500). HT22 cells (a mouse hippocampal cell line) were a kind gift from Professor Tillman Grune (Friedrich Schiller University, Jena, Germany) and cultured in Dulbecco’s modified Eagle’s medium (DMEM) supplemented with 10% fetal bovine serum, 100 U/mL penicillin, and 100 μg/mL streptomycin B. CPAE cells (an endothelial cell line derived from the bovine stem pulmonary artery) were obtained from the American Type Culture Collection (ATCC CCL 209, Rockville, MD, USA) and grown in minimum essential Eagle’s medium supplemented with 20% fetal bovine serum, 100 U/mL penicillin, and 100 μg/mL streptomycin B. The cells were maintained at 37 °C under an atmosphere of 95% air and 5% CO_2_. For the experiments, the cells were seeded in a plate in an adequate amount and allowed to attach overnight. The following day, the cells were treated with various combination of 0.4 μmol/L Ang II, 20 μmol/L SP600125, and 25 μmol/L deferoxamine (DFO), as indicated.

### 2.2. Immunoblotting

The cells were treated as described in Section 2.1. Both floating and attached cells were collected on the same day, washed in PBS, resuspended in a lysis solution containing 50 mmol/L Tris-HCl (pH 7.5), 150 mmol/L NaCl, 1% (*v*/*v*) Triton X-100, 0.1% (***w****/v*) SDS, and incubated for 40 min on ice with gentle shaking. The cell lysate was cleared by centrifugation at 16,000 × *g* for 20 min. Proteins in the lysate were resolved on 10%–12% SDS-PAGE gel. The proteins were then transferred onto a PVDF membrane by using a standard semi-dry technique. After blocking in a 5% (*w/v*) solution of dried skim milk in TBS buffer containing 0.1% (*v/v*) Tween 20, the membrane was incubated with a specific primary antibody, as appropriate, overnight at 4 °C. The membrane was then treated with an appropriate secondary antibody, and immunoreactive bands were visualized using enhanced chemiluminescence (Perkin Elmer ECL Plus, Waltham, USA). Changes in protein levels were evaluated by densitometric analysis and normalized to the β-actin loading control. The following antibodies were used: anti-ferritin H antibodies: Santa Cruz Biotechnology (sc-25617) (1:500) and Cell Signalling Technology (#3998) (1:1000), anti-ferritin L antibodies: Santa Cruz Biotechnology (sc-74513) (1:1000) and abcam (ab69090) (1:1000), anti-phospho-p66Shc (Ser 36) antibodies: abcam (ab545118) (1:1000), anti-Shc (H-108) antibodies: Santa Cruz Biotechnology (sc-1695) (1:1000); anti-β-actin antibodies: Sigma Aldrich (A3854) (1:50000), HA-probe (F7): Santa Cruz Biotechnology (sc-7392) (1:1000), and anti-ITCH antibodies: Life Span Biosciences (ERP4936) (1:500), anti-rabbit IgG antibodies: Sigma Aldrich (1:25000), anti-mouse IgG antibodies Sigma Aldrich (1:250000).

### 2.3. Transient Transfection

Plasmid encoding a p66Shc variant (p66ShcS36A) was kindly provided by Dr. Toren Finkel (NIH Bethesda). Plasmids encoding catalytically inactive variants of ITCH (ITCH-AA), JNK 1, and JNK2 were kindly provided by Dr. Michael Karin (University of California, San Diego). CPAE cells were transfected with the plasmids encoding catalytically inactive variants of JNK1 and JNK2, ITCH-AA, or p66ShcS36A, or an empty pcDNA3.1 vector at 50%–60% confluency using polyethylenimine PEI (Sigma Aldrich). Then, 24 h after transfection, the cells were treated with Ang II and assayed as described.

### 2.4. Determination of LIP

For determination of LIP, the calcein fluorescence method was used. CPEA cells were seeded at a density of 5 × 10^5^ per 60-mm dish, allowed to attach, and exposed to Ang II for 1 h in the absence or presence of 25 μmol/L deferoxamine. The cells were then stained with 5 μmol/L calcein acetoxymethyl AM for 30 min, collected by trypsinization and centrifugation, washed two times in PBS, and analyzed immediately using a confocal microscope. Cell images were analyzed using the ImageJ program (National Institutes of Health and the Laboratory for Optical and Computational Instrumentation, Wisconsin, USA.

### 2.5. Measurement of ROS Levels

Intracellular ROS generation was determined by flow cytometric monitoring of the oxidation of 2’,7’-dichlorodihydrofluorescein diacetate (H_2_DCFDA). The compound is cleaved by nonspecific cellular esterases and oxidized in the presence of peroxides and iron. For the experiment, 2 × 10^5^ cells were plated, allowed to attach overnight, and exposed to 0.4 μM Ang II for 1 h. Subsequently, the cells were stained with 5 μmol/L H_2_DCFDA for 30 min at 37 °C in a complete medium. The cells were collected by trypsinization and centrifugation, washed two times in PBS, and analyzed immediately using a Coulter Epics XL flow cytometer. The fluorescence of unstained cells was also measured, to determine background fluorescence.

### 2.6. Statistical Analysis

Statistical analyses were performed using Graphpad Prism. Data were presented as mean ± SEM. The differences between the means were determined by the Student’s t-test or one-way analysis of variance (ANOVA). The post hoc Tukey’s or Dunnett’s multiple comparison tests were performed to identify significantly different groups. The significance level *p* < 0.05 was accepted for analysis.

## 3. Results

### 3.1. Angiotensin II Induces Ferritin Degradation and LIP Increase

**Figure 1:**
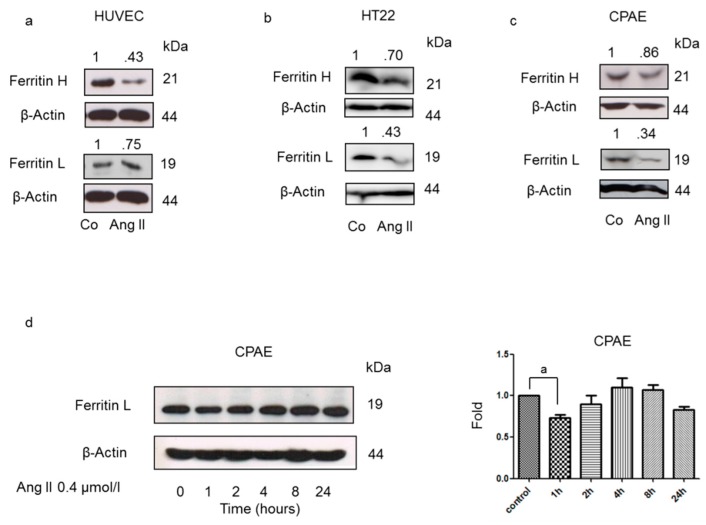
Angiotensin II (Ang II) induces ferritin degradation. Immunoblotting analysis of ferritin H and L levels in lysates of (**a**) human umbilical vein endothelial cells (HUVEC), (**b**) HT22, and (**c**) CPAE cells treated with 0.4 μmol/L Ang II for (**a**) 4 h, (**b**) 1 h, and (**c**) 1 h. After probing with specific antibodies, the blots were stripped and reprobed with an anti-β-actin antibody to normalize for differences in protein loading. The results are representative of three independent experiments. (**d**) Immunoblotting analysis of ferritin L levels in a lysate of CPAE cells treated with 0.4 μmol/l Ang II for the indicated time periods. After probing with specific antibodies, the blots were stripped and reprobed with an anti-β-actin antibody to normalize for differences in protein loading. Data in the graph on the right are the mean ± SEM from three independent experiments, with values recalculated relative to the control; “a” indicates statistical significance at *p* < 0.05 (t-test).

Ferritin is the main cellular iron storage protein. A reduction of cellular ferritin levels leads to an increase of chelatable iron levels. We first aimed to establish whether Ang II influences ferritin H and L protein levels in HUVEC, HT22, and CPAE cells. As shown in Figure 1a–c, Ang II induced a pronounced drop in cellular ferritin H and L protein levels. To obtain insight into the mechanism of the observed ferritin level reduction, all other experiments were performed using the CPAE cells. As shown in Figure 1d, Ang II-induced ferritin degradation was most pronounced after 1–2 h of Ang II treatment.

**Figure 2:**
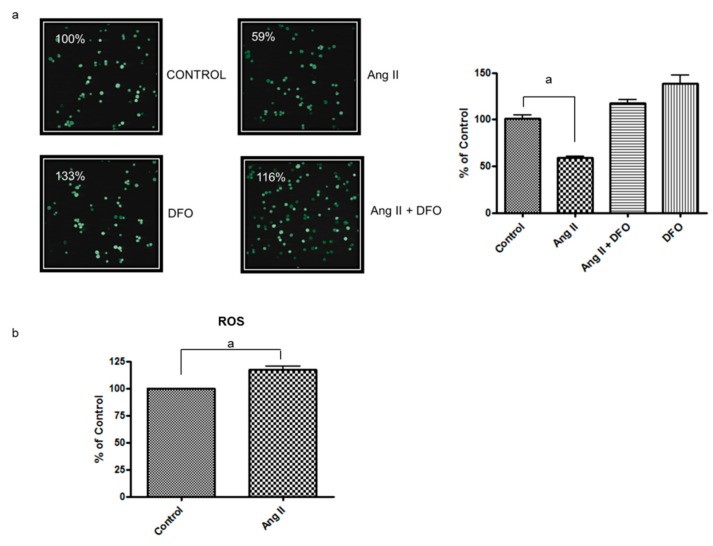
Ang II-induced reactive oxygen species (ROS) generation is associated with labile iron pool (LIP) increase. (**a**) Representative images of confocal microscopy analysis of calcein fluorescence of CPAE cells treated with 0.4 μmol/L Ang II. CPAE cells were pretreated with 25 μmol/L DFO for 1 h. The graph on the right shows the percentage of cells exhibiting quenched fluorescence. Data are shown as the mean + SEM from four independent experiments, recalculated relative to the control: “a” indicates statistical significance at *p* < 0.001 (one-way ANOVA followed by Tukey’s multiple comparison test); (**b**) Percent of control DCF fluorescence in CPAE cells treated for 1 h with 0.4 μmol/L Ang II. Data are presented as the mean + SE (*n* = 3); “a” indicates statistical significance at *p* < 0.022 (t-test).

It has been reported that the decrease in ferritin H levels leads to an increase in LIP [21]. Hence, the changes in LIP were next evaluated in CPAE cells treated with Ang II by using fluorescence microscopy, following cell staining with calcein, an iron-sensitive probe. As shown in Figure 2a, approximately 41% of the CPAE cells treated with Ang II exhibited quenched calcein fluorescence compared with the control, indicating an increase in LIP. DFO, a specific iron chelator, fully reversed the effect of Ang II, confirming the LIP increase by Ang II treatment. A LIP increase is usually accompanied by an increase in ROS formation. Indeed, stimulation ofH_2_DCFDA -loaded CPAE cells with Ang II (0.4 μmol/L) resulted in increased 2’,7’- dichlorofluoresceina (DCF) fluorescence (Figure 2b).

### 3.2. Ferritin Degradation Induced by Ang II is Mediated by JNK

**Figure 3:**
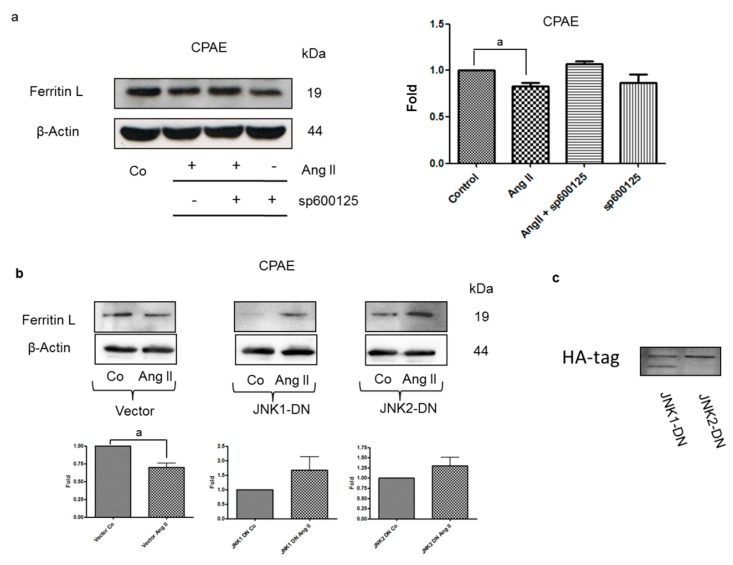
Ang II-induced ferritin degradation is c-Jun terminal kinase (JNK) dependent. (**a**) Immunoblotting analysis of ferritin L levels in lysates of control CPAE cells or cells treated for 1 h with Ang II in the absence or presence of 20 μmol/L SP600125 (1 h pretreatment). After probing with specific antibodies, the blots were stripped and reprobed with an anti-β-actin antibody to normalize for differences in protein loading. Data in the graph on the right are the mean + SEM from three independent experiments, recalculated relative to the control; “a” indicates statistical significance at *p* < 0.05 (t-test). (**b**) Immunoblotting analysis of ferritin L levels in lysates of CPAE cells transiently transfected with an empty vector, or vectors encoding catalytically inactive variants of JNK1 or JNK2 following a 1-h treatment with 0.4 μmol/L Ang II. After probing with specific antibodies, the blots were stripped and reprobed with an anti-β-actin antibody to normalize for differences in protein loading. Data in the graphs at the bottom of the panel are the mean ± SEM from three independent experiments, recalculated relative to the control; “a” indicates statistical significance at *p* < 0.05 (t-test). (**c**) Immunoblotting detection of the HA-tag in lysates of CPAE cells transiently transfected with vectors encoding HA-tagged catalytically inactive variants of JNK1 or JNK2.

The JNK signaling axis regulates ferritin degradation and iron-dependent ROS formation [20]. To establish whether Ang II-mediated ferritin degradation is JNK dependent, the CPAE cells were treated with a JNK inhibitor, SP600125. As shown in Figure 3a, SP600125 completely blocked ferritin degradation induced by Ang II. To confirm the involvement of JNK in ferritin degradation, the cells were next transfected with vectors expressing the dominant negative catalytically inactive variants of JNK1 (JNK1-DN) or JNK2 (JNK2-DN). Ang II treatment caused ferritin degradation in cells transfected with an empty vector, while ferritin L levels in cells transfected with vectors expressing JNK1-DN or JNK2-DN were not affected. Collectively, these observations indicate that Ang II-induced ferritin degradation depends on JNK.

### 3.3. Ferritin Degradation is Mediated by ITCH

**Figure 4:**
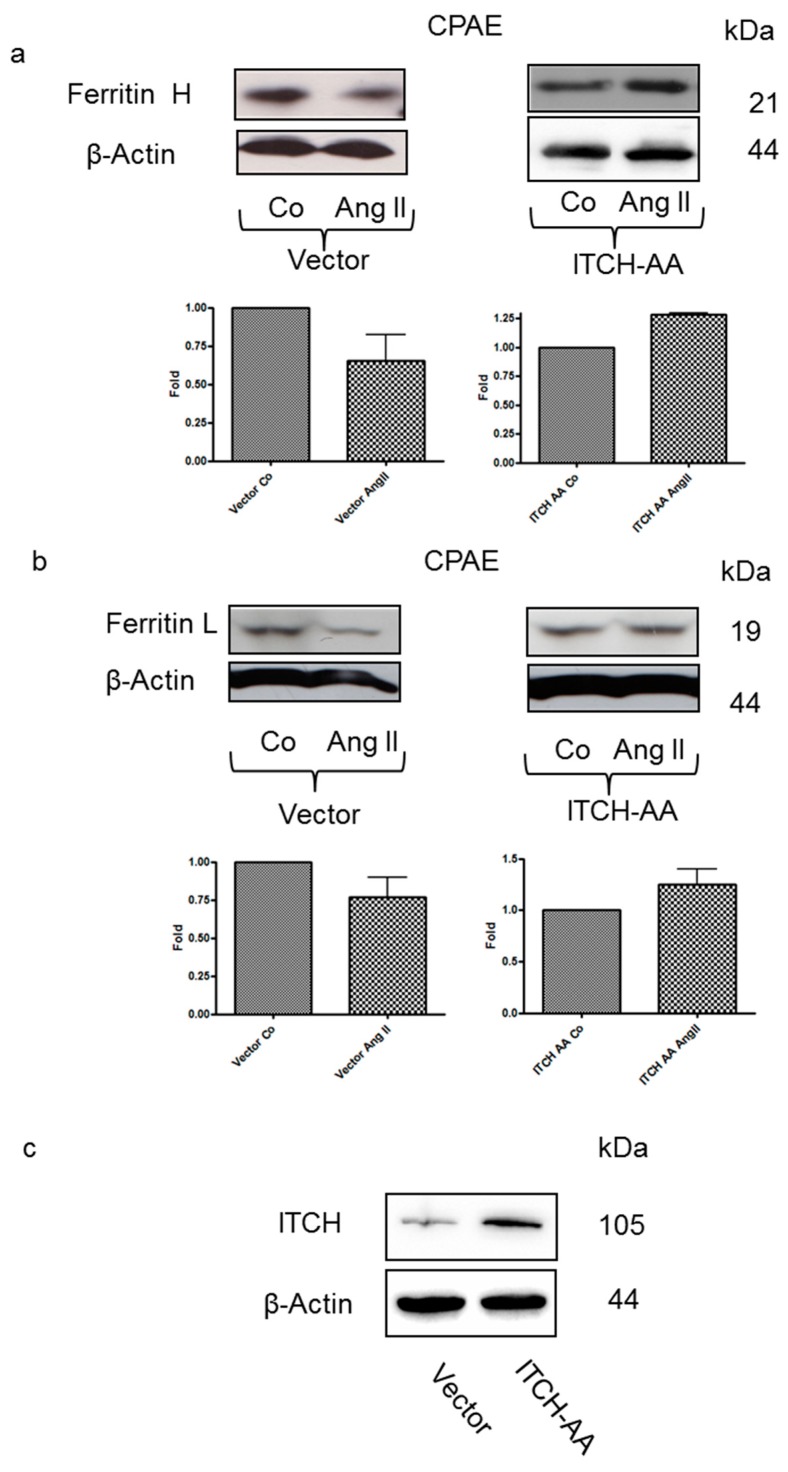
Ang II-induced ferritin degradation is ITCH-dependent. Immunoblotting analysis of (**a**) ferritin H and (**b**) L levels in lysates of CPAE cells transiently transfected with an empty vector or a vector encoding a catalytically inactive mutant of ITCH following a 1-h treatment with 0.4 μmol/L Ang II. After probing with specific antibodies, the blots were stripped and reprobed with an anti-β-actin antibody to normalize for differences in protein loading. Data in graphs at the bottom of the panel are the mean + SEM from three independent experiments, recalculated relative to the control. (**c**) Immunoblotting analysis of the ITCHlevels in lysates of CPAE cells transiently transfected with an empty vector or a vector encoding a catalytically inactive variant of ITCH. After probing with specific antibodies, the blots were stripped and reprobed with an anti-β-actin antibody to normalize for differences in protein loading.

JNK1 regulates degradation of some proteins by phosphorylation of the ubiquitin ligase E3 ITCH. Hence, we hypothesized that JNK-dependent ferritin degradation might be mediated by ITCH. To determine whether ITCH plays a role in Ang II-induced ferritin degradation, cells were transfected with a vector expressing an inactive variant of ITCH (ITCH-AA). As shown in Figure 4, Ang II treatment caused ferritin degradation in cells transfected with an empty vector, while ferritin L and H levels in cells transfected with a vector expressing ITCH-AA were slightly increased.

### 3.4. Ang II-induced Ferritin Degradation is Mediated by p66Shc

**Figure 5:**
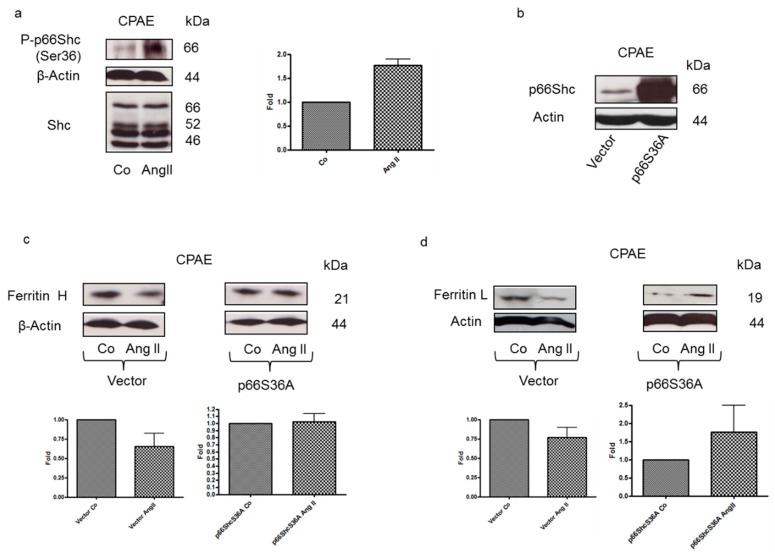
Ang II-induced ferritin degradation is p66Shc dependent. (**a**) Immunoblotting analysis of p66Shc and p-p66Shc levels in lysates of control CPAE cells or cell treated for 1 h with Ang II. After probing with specific antibodies, the blots were stripped and reprobed with an anti-β-actin antibody to normalize for differences in protein loading. Data in the graphs on the right are the mean + SEM from two independent experiments, recalculated relative to the control. (**b**) Immunoblotting analysis of p66Shc levels in lysates of CPAE cells transiently transfected with an empty vector or a vector encoding a dominant negative variant of p66Shc (p66ShcS36A). Immunoblotting analysis of (**c**) ferritin H and (**d**) ferritin L levels in lysates of CPAE cells transiently transfected with an empty vector or a vector encoding a dominant negative variant of p66Shc (p66ShcS36A) following a 1-h treatment with 0.4 μmol/L Ang II. After probing with specific antibodies, the blots were stripped and reprobed with an anti-β-actin antibody to normalize for differences in protein loading. Data in graphs at the bottom of the panel are the mean + SEM from three independent experiments, recalculated relative to the control.

Protein p66Shc stimulates ROS formation after it is phosphorylated on serine 36. In the current study, a rise in serine-phosphorylated p66Shc levels was apparent in cells treated with Ang II (Figure 5a). Previously, we demonstrated that p66Shc phosphorylated on serine 36 stimulates ferritin degradation and iron-dependent ROS formation in prostate cancer cell [18]. To check whether such a mechanism is also operational in CPAE cells, the cells were transfected with a plasmid encoding a dominant negative variant of p66Shc (p66ShcS36A) (Figure 5b). Both ferritin L and H degradation induced by Ang II were significantly reduced in CPAE cells expressing p66ShcS36A compared with cells transfected with an empty vector (Figure 5c,d).

## 4. Discussion

In the current study, we aimed to demonstrate that Ang II treatment leads to a disturbance of iron metabolism by increasing ferritin degradation and releasing iron.

The effect of Ang II on iron metabolism has been demonstrated in several studies. For example, it has been observed that Ang II induces iron accumulation in the aorta, brain, and heart [22]. Iron is involved in the pathophysiology of abdominal aortic aneurysm (AAA), with oxidative stress and inflammation, and dietary iron restriction inhibits Ang II–induced AAA formation [22]. This is somewhat puzzling since iron is stored in ferritin, which protects it from participating in ROS-generating reactions. Several years ago, Sullivan proposed that intracellularly stored iron is not inert [15]. However, the molecular mechanism of its toxicity has not yet been fully explored. In the present study, we demonstrated that Ang II induces ferritin degradation in the neuronal and endothelial cell lines, and that this process is controlled by the JNK/p66Shc/ITCH signaling pathway.

Iron can stimulate intracellular ROS formation and is present in a chelatable iron pool and LIP [23]. A LIP is defined as a redox-active iron pool, chelated by low-molecular weight compounds. Because of its high toxicity, a LIP is low and strictly regulated [24]. Although ferritin stores iron and prevents its participation in ROS-generating reactions, it can also be a source of a LIP. The amount of ferritin-bound iron is much higher than that in a LIP. Hence, iron liberation from ferritin can significantly increase a LIP. In addition, upregulation of ferritin levels decreases cellular LIP, while downregulation of ferritin levels has the opposite effect.

In the current study, we unambiguously demonstrated that Ang II enhances ferritin degradation and that this is accompanied by LIP increase and ROS formation. Knowledge of the molecular mechanism responsible for ferritin degradation could facilitate the understanding of the pathomechanism of several diseases related to excess iron accumulation. Ferritin undergoes proteolytic degradation in the lysosome and proteasome, which is associated with LIP increase [25,26]. However, the molecular mechanism controlling ferritin degradation is not fully understood. It has been reported that the human AAA tissue contains high levels of activated JNK and that inhibition of JNK in vivo prevents the development of AAA in a mouse model [27,28]. Indeed, we have previously observed that proteasomal ferritin degradation in cancer cells is mediated by JNK1, a SAPK [19]. Here we observed that Ang II-induced ferritin degradation was abrogated in cells pre-treated with JNK inhibitor or expressing JNK-DN, however the role of proteasome needs to be further studied. JNK kinases are multifunctional and have many substrates, including the E3 ubiquitin ligase ITCH. ITCH is activated by JNK1, which leads to cFLIP ubiquitination and stimulates its proteasomal degradation [29]. Here, we observed that Ang II-induced ferritin degradation was abrogated in CPAE cells expressing an inactive form of ITCH. These observations strongly support the involvement of this ligase in ferritin degradation.

Another substrate of JNK is p66Shc, an adaptor protein involved in mitochondrial ROS formation and modulation of several signaling pathways [30,31]. It has been shown that p66Shc mediates ROS formation when phosphorylated on serine 36 [32]. Inactivation of p66Shc protects against endothelial dysfunction and oxidative stress induced by age-related hypercholesterolemia and hyperglycemia [33,34]. In addition, trichostatin A treatment inhibits vasoconstriction and hypertension by inhibiting Ang II-induced phosphorylation of p66Shc [35]. In the current study, we demonstrated that Ang II-induced ferritin H and L degradation is mediated by p66Shc phosphorylated on serine 36.

Collectively, the presented data indicate that Ang II can increase LIP in endothelial and other cell lines, and that this process is mediated by the JNK/p66Shc/ITCH signaling pathway. Ang II, a potent activator of NADPH oxidases, decreases the bioavailability of NO by stimulating excessive ROS formation (especially superoxide formation). Ang II-induced LIP increase may lead to the formation of the hydroxyl radical but also to a decrease of NO levels. It has been demonstrated that NO reacts with LIP, similar to the strong iron chelator salicylaldehyde isonicotinoyl hydrazone [36]. This reaction produces dinitrosyl iron complex, a form of iron unable to react with hydrogen peroxide that gives rise to hydroxyl radical [37]. Hence, formation of the NO-iron complex may have profound consequences for cellular metabolism. On the one hand, reduced iron-dependent ROS formation reduces the oxidative damage to cellular macromolecules [37]. On the other hand, it can be expected that in cells enriched in ferritin iron, Ang II-enhanced ferritin degradation and increased LIP would lead to a decreased bioavailability of ·NO. Consistently, aortic stiffness and vascular remodeling in chronically iron-overloaded rats is reversed by Losartan, an antagonist of the Ang II receptor [38].

## 5. Conclusions

**Figure 6:**
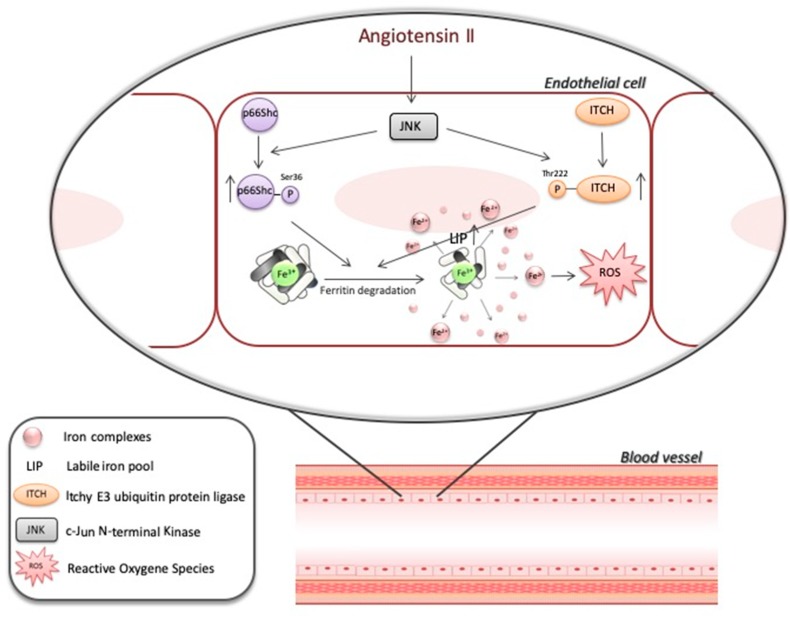
Cell exposure to Ang II leads to ferritin degradation. This process is an outcome of p66Shc protein and ubiquitin ligase ITCH activation, which depends on JNK. As a consequence of ferritin degradation, LIP and iron-dependent ROS formation both increase.

The data presented herein indicate that the JNK/p66Shc/ITCH signaling pathway modulates the intracellular LIP. As the JNK/p66Shc/ITCH pathway is part of the cell response to stress, including exposure to high Ang II levels, more studies are needed to explore the role of iron in this response (Figure 6).
